# Supplementary material for: Monoclonal antibodies from humans with Mycobacterium tuberculosis exposure or latent infection recognize distinct arabinomannan epitopes
Source: Commun Biol. 2021 Oct 12;4:1181. doi: 10.1038/s42003-021-02714-w (PMC8511196; doi:10.1038/s42003-021-02714-w)
Supplement: Supplementary file 2 — Supplementary Information [file 42003_2021_2714_MOESM2_ESM.pdf]

Supplementary Figures:

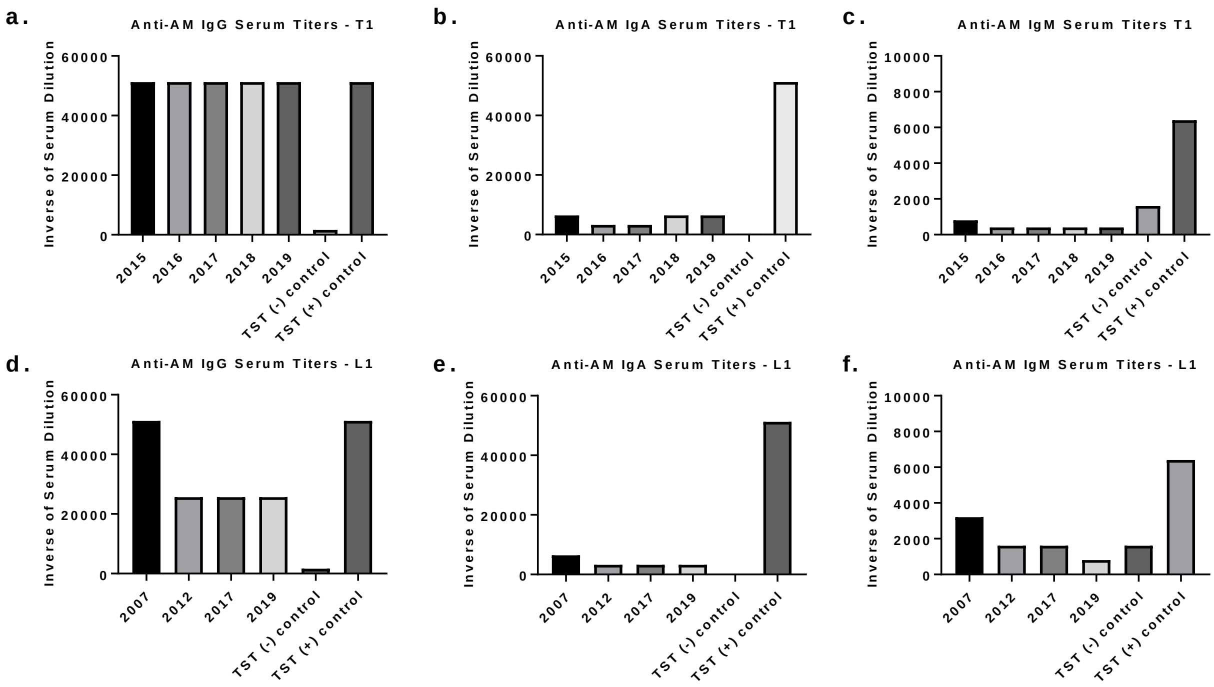

Supplementary Figure 1. Anti-AM immunoglobulin titers in sera of subjects T1 and L1 over several years. Anti-AM IgG, IgA, and IgM titers from subject T1 **(a-c)** and subject L1 **(d-f)** by ELISA. Endpoint titers were defined as having an optical density at least 3 standard deviations above background after subtraction of nonspecific (BSA) signal.

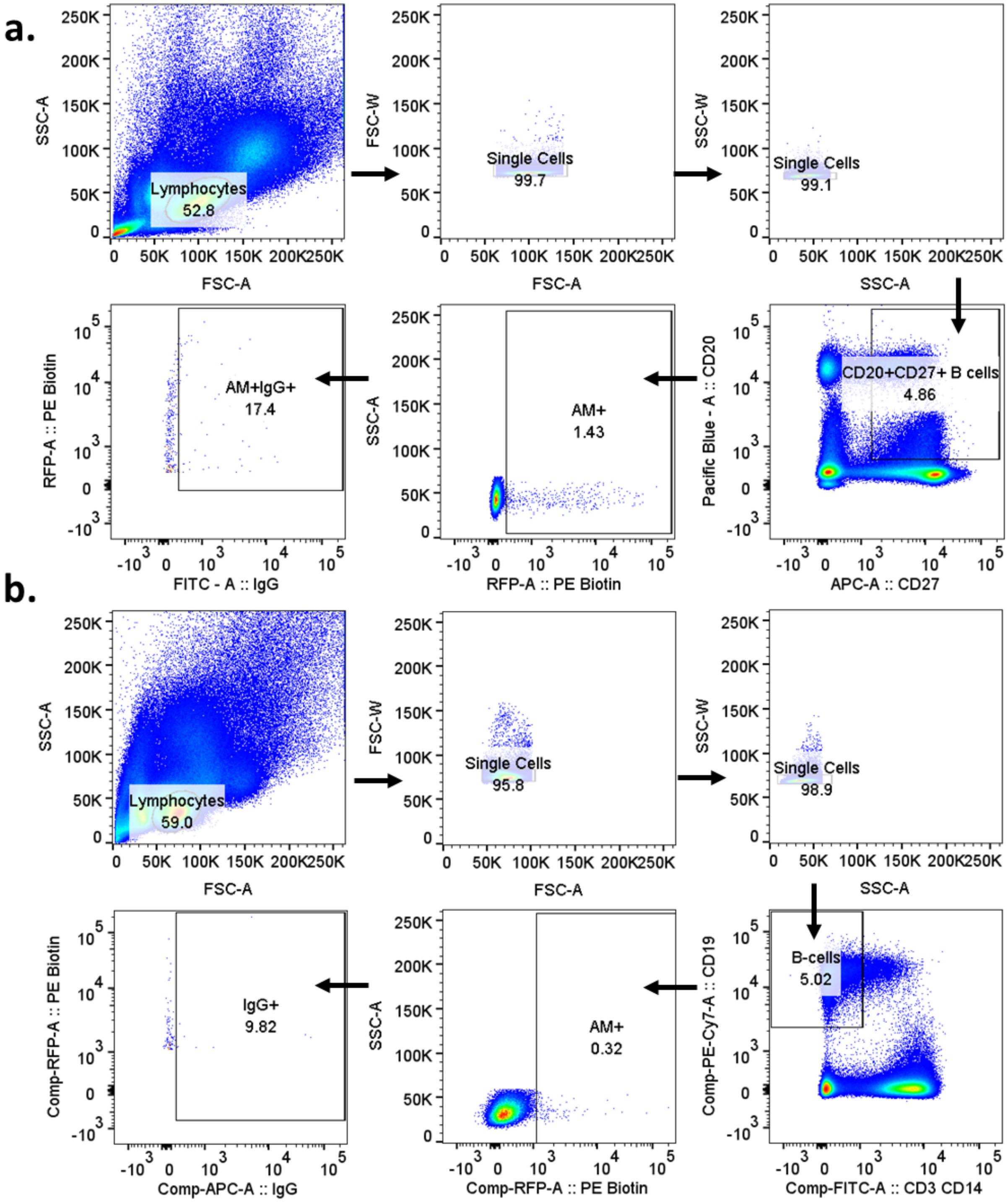

Supplementary Figure 2. Anti-AM specific human B cells sorting strategies.

(a) Gating of CD20+, CD27+, AM+, IgG+ memory B cells from a sample of peripheral blood mononuclear cells (PBMC) collected from subject T1. **(b)** Gating of CD19+, AM+, IgG+, B cells from a sample of PBMC collected from subject L1.

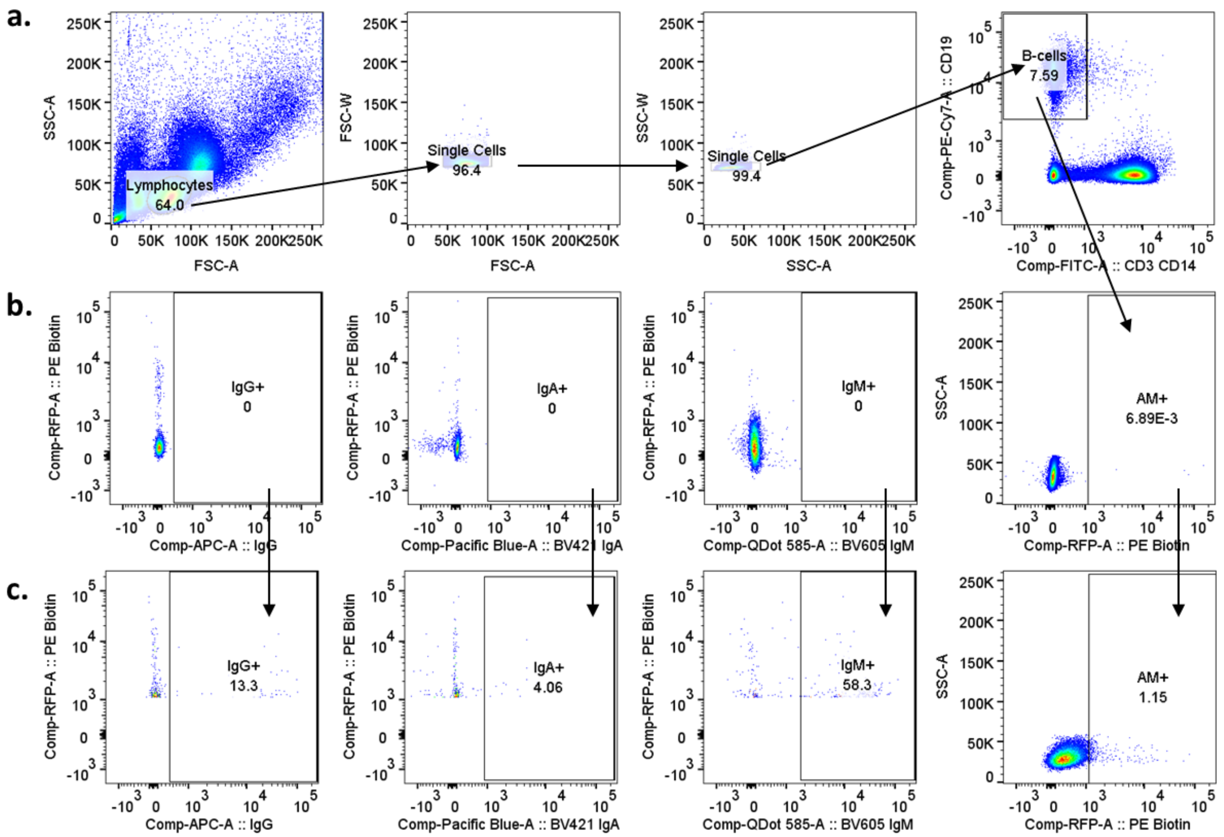

Supplementary Figure 3. Representative gating strategy using Fluorescence minus one (FMO) controls (a) Lymphocytes and single cells are gated, followed by live B cells. **(b)** Fluorescence minus one (FMO) controls are shown for PE (biotinylated AM), APC (IgG), BV421 (IgA), and BV605 (IgM). **(c)** Frequency of anti-AM human B cells from Subject T1.

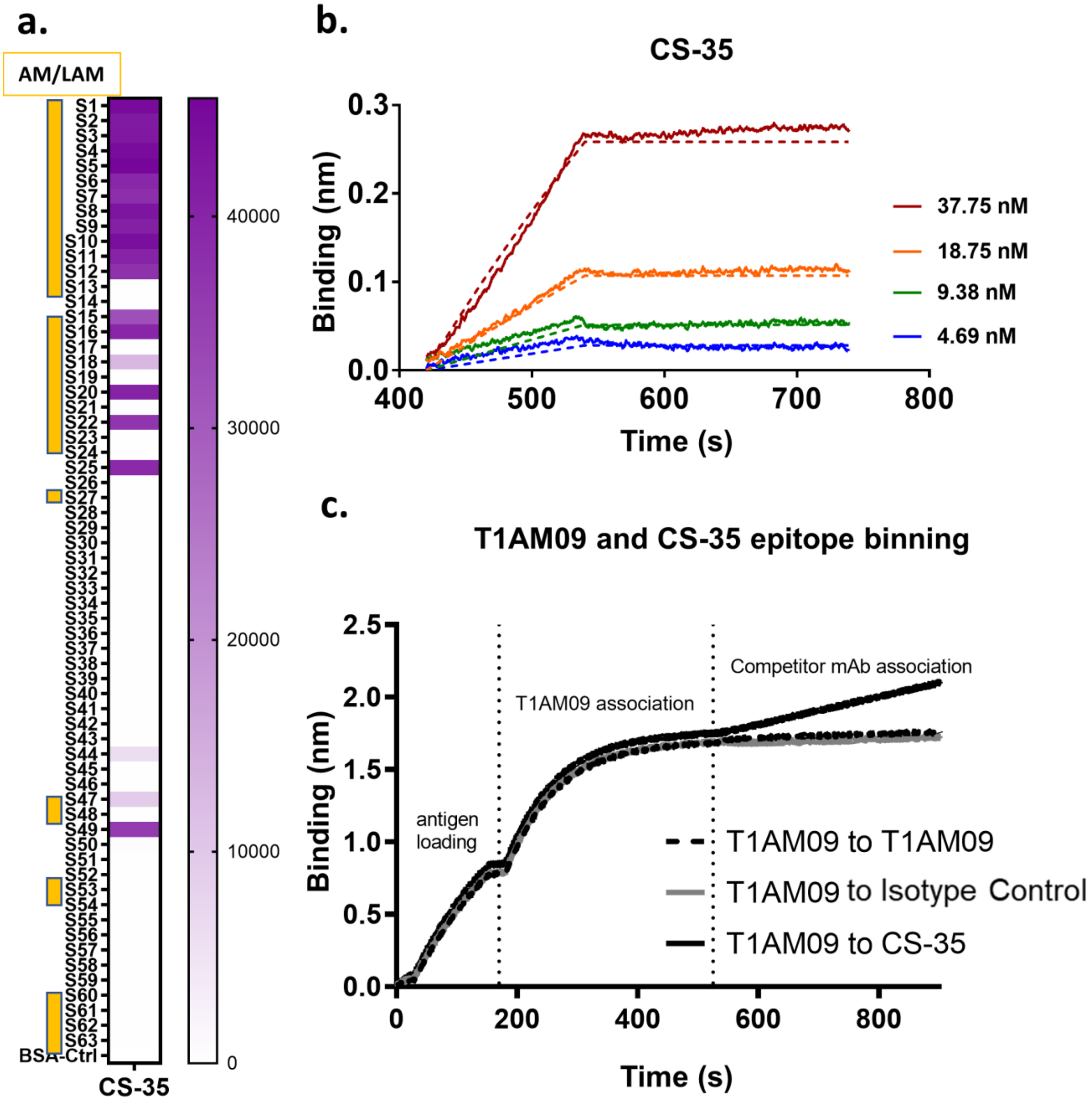

Supplementary Figure 4. Murine anti-LAM mAb CS-35 has distinct binding characteristics from human anti-AM mAbs T1AM09 and L1AM04. (a) CS-35 glycan fingerprint; arabinomannan/lipoarabinomannan (AM/LAM) specific fragments (S#1-12, 15-22, 25, 44, 45, 49, 50, 56-59) are marked by the yellow side bar. Six other glycans on the array are: α-glucan (S#13, 14, 24, 46, 48, 52), trehalose mycolates and lipooligosachharides (LOSs; S#38, 39, 54, 55), phenolic glycolipids (PGLs; S#26-37, 40-43, 50, 53), phosphatidyl-myo-inositol mannosides (PIMs, S#23) and glycopepitdolipids (GPLs; S#47, 60, 61). **(b)** Binding curve of CS-35 binding to biotinylated AM generated by BLI solid lines represent experimental data and dashed lines are statistically fitted curves; $K_{D, app}$: 1.0 E-12 M **(c)** Two-phase binding experiment detecting T1AM09 competition with CS-35. Binding to self (T1AM09) and to isotype control mAb are negative controls.

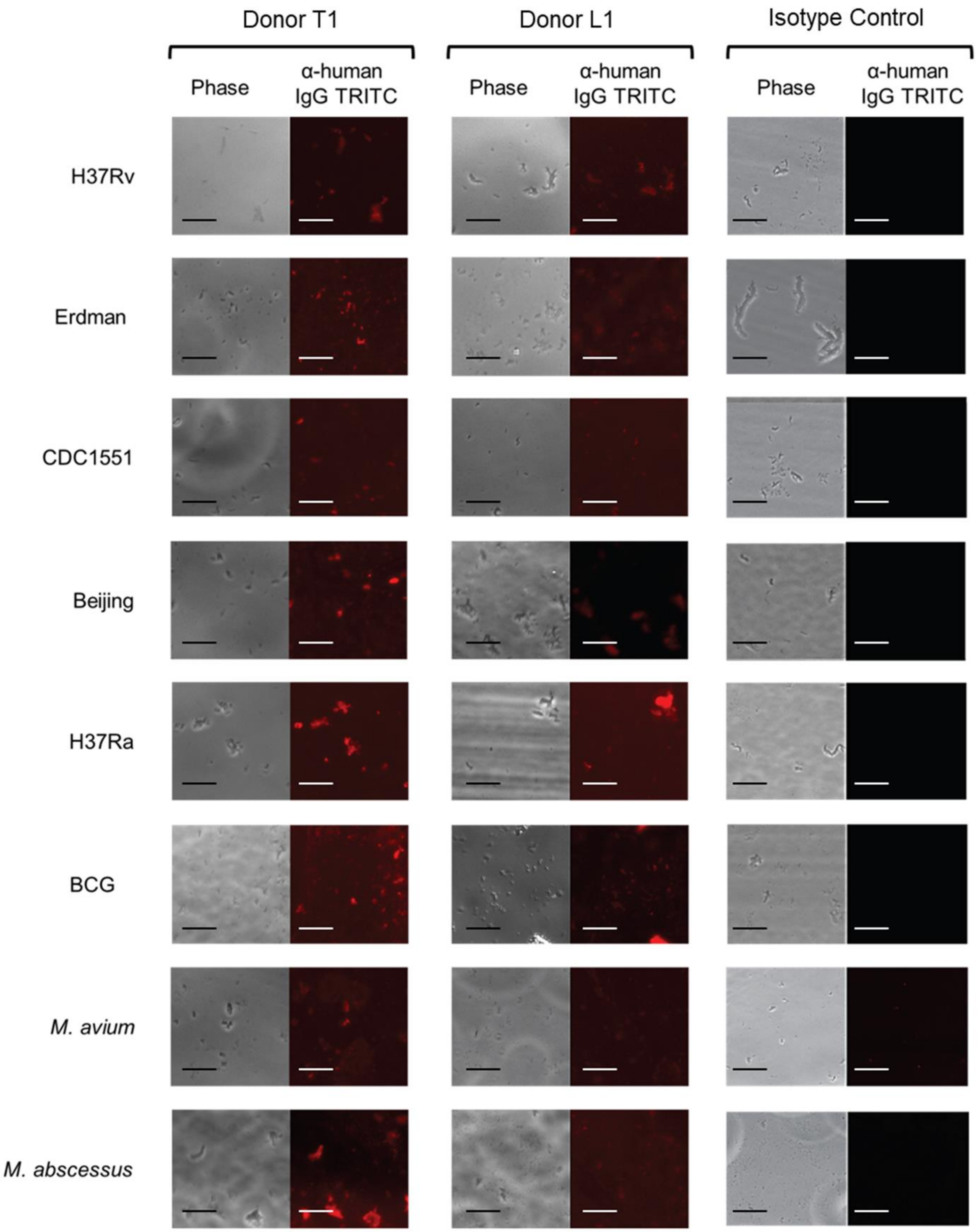

Supplementary Figure 5. Binding of donor sera (T1 and L1; 1:100) and negative control (human IgG1; 10 μg/ml) to mycobacteria strains (scale bar 10 μm).

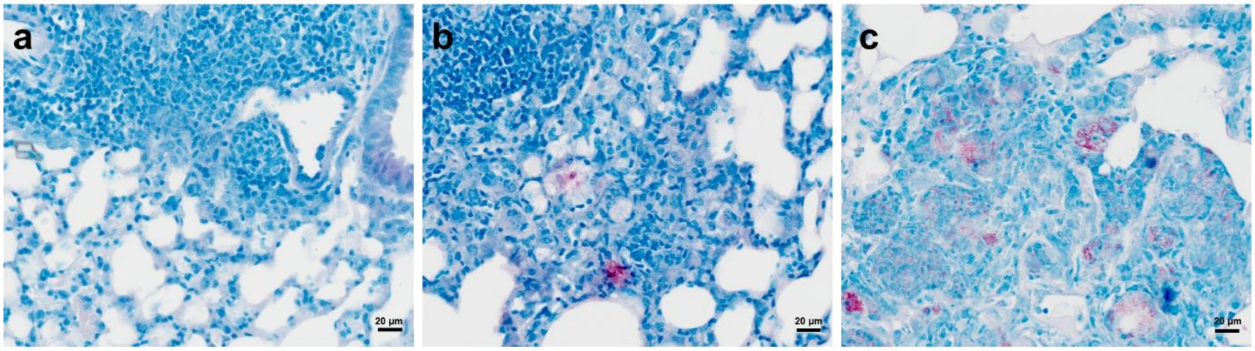

Supplementary Figure 6. Fite's Method lacks sensitivity for *Mtb* CDC1551 and Erdman in murine infected tissue. (a) AFB staining of murine lungs infected with *Mtb* (CDC1551); **(b)** AFB staining of murine lungs murine lungs infected with *Mtb* (Erdman) arrow – indicates only positive staining in the lung; **(c)** *Mtb*-infected murine lung tissue provided by the Einstein Histology and Pathology Core was used as a positive control for Fite's method (scale bar 20 µm).

Supplementary Tables:

Supplementary Table 1. Characteristics of heavy and kappa chain and percent nucleotide identity for mAbs generated from subject T1

Antibody ID	Heavy V Gene	CDRH3 Length (AA)	VH Identity (nt %)	Kappa V Gene	CDRL3 Length (AA)	VK Identity (nt %)
**T1AM05**	5-10-1*01	13	92.71	1-5*03	9	94.98
**T1AM06**	2-15*01	14	90.14	3-20*01	10	92.31
**T1AM07**	3-23*01	16	88.19	3-20*01	8	87.23
**T1AM09**	1-2*02	14	91.32	1-39*01	9	89.96
**T1AM11**	3-66*01	8	91.58	1-33*01	8	89.96
**T1AM13**	3-7*01	21	93.06	4-2*01	10	95.96
**T1AM16**	1-2*02	16	96.18	3-11*01	10	95.70
**T1AM39**	3-30-3*01	10	96.18	3-20*01	11	92.11
**T1AM47**	3-7*02	14	95.49	3-20*01	8	98.21
Average % nt identity:			92.76%			92.94%

Supplementary Table 2. Characteristics of heavy and kappa chain and percent nucleotide identity for mAbs generated from subject L1

Antibody ID	Heavy V Gene	CDRH3 Length (AA)	VH Identity (nt %)	Kappa V Gene	CDRL3 Length (AA)	VK Identity (nt %)
**L1AM01**	1-24*01	11	94.44	4-1*01	9	91.67
**L1AM03**	1-3*01	12	96.53	1-9*01	9	98.21

**L1AM04**	3-23*01	13	92.01	2-24*01	9	91.84
**L1AM05**	3-15*07	12	95.58	3-20*01	9	95.39
**L1AM08**	4-4*07	14	95.44	1-39*01	9	90.68
**L1AM10**	4-4*07	14	95.79	1-9*01	9	98.21
**L1AM13**	3-21*01	16	94.79	2-24*01	9	91.84
**L1AM14**	3-15*07	12	95.58	3-20*01	9	95.39
Average % nt identity:			95.02			94.15

Supplementary Table 3. Maximum and effective concentrations of mAbs binding to arabinomannan (AM) from different mycobacterial strains

	T1AM09		L1AM04	
	**Maximum OD**	**Average EC50 (µg/ml)**	**Maximum OD**	**Average EC50 (µg/ml)**
RvAM biotin	2.0 ± 0.10	0.1 ± 0.03	2.2 ± 0.10	0.9 ± 0.27
H37Rv AM	1.9 ± 0.28	0.1 ± 0.04	2.1 ± 0.20	1.0 ± 0.37
H37Ra AM	1.9 ± 0.06	0.1 ± 0.01	1.3 ± 0.32	6.4 ± 5.17
CDC1551 AM	1.9 ± 0.18	0.1 ± 0.03	1.9 ± 0.40	1.4 ± 0.18
Beijing AM	1.9 ± 0.12	0.1 ± 0.04	1.8 ± 0.34	0.9 ± 0.18
BCG AM	1.9 ± 0.19	0.1 ± 0.03	1.1 ± 0.32	9.9 ± 8.61
p-value (group comparison)	0.988*	0.312*	0.003*	0.015^

Optical density (OD_{450}) ± standard deviation (SD) and effective concentrations (EC50) ± SD of mAbs (ug/mL) binding to arabinomannan (AM) from mycobacterial strains (*ANOVA; ^Kruskal-Wallis)
