# Supplementary material for: Monoclonal antibodies from humans with Mycobacterium tuberculosis exposure or latent infection recognize distinct arabinomannan epitopes
Source: Commun Biol. 2021 Oct 12;4:1181. doi: 10.1038/s42003-021-02714-w (PMC8511196; doi:10.1038/s42003-021-02714-w)
Supplement: Supplementary file 3 — Reporting Summary [file 42003_2021_2714_MOESM3_ESM.pdf]

nature research

Corresponding author(s): Jacqueline M. Achkar

Last updated by author(s): 7/21/2021

Reporting Summary

Nature Research wishes to improve the reproducibility of the work that we publish. This form provides structure for consistency and transparency in reporting. For further information on Nature Research policies, see our Editorial Policies and the Editorial Policy Checklist.

Statistics

For all statistical analyses, confirm that the following items are present in the figure legend, table legend, main text, or Methods section.

n/a	Confirmed	
☐	☒	The exact sample size (*n*) for each experimental group/condition, given as a discrete number and unit of measurement
☐	☒	A statement on whether measurements were taken from distinct samples or whether the same sample was measured repeatedly
☐	☒	The statistical test(s) used AND whether they are one- or two-sided<br>*Only common tests should be described solely by name; describe more complex techniques in the Methods section.*
☒	☐	A description of all covariates tested
☒	☐	A description of any assumptions or corrections, such as tests of normality and adjustment for multiple comparisons
☐	☒	A full description of the statistical parameters including central tendency (e.g. means) or other basic estimates (e.g. regression coefficient) AND variation (e.g. standard deviation) or associated estimates of uncertainty (e.g. confidence intervals)
☐	☒	For null hypothesis testing, the test statistic (e.g. *F*, *t*, *r*) with confidence intervals, effect sizes, degrees of freedom and *P* value noted<br>*Give P values as exact values whenever suitable.*
☒	☐	For Bayesian analysis, information on the choice of priors and Markov chain Monte Carlo settings
☒	☐	For hierarchical and complex designs, identification of the appropriate level for tests and full reporting of outcomes
☒	☐	Estimates of effect sizes (e.g. Cohen's *d*, Pearson's *r*), indicating how they were calculated

Our web collection on statistics for biologists contains articles on many of the points above.

Software and code

Policy information about availability of computer code

Data collection	*Provide a description of all commercial, open source and custom code used to collect the data in this study, specifying the version used OR state that no software was used.*
Data analysis	*Provide a description of all commercial, open source and custom code used to analyse the data in this study, specifying the version used OR state that no software was used.*

For manuscripts utilizing custom algorithms or software that are central to the research but not yet described in published literature, software must be made available to editors and reviewers. We strongly encourage code deposition in a community repository (e.g. GitHub). See the Nature Research guidelines for submitting code & software for further information.

Data

Policy information about availability of data

All manuscripts must include a data availability statement. This statement should provide the following information, where applicable:

- Accession codes, unique identifiers, or web links for publicly available datasets
- A list of figures that have associated raw data
- A description of any restrictions on data availability

The accession numbers for the nucleotide and protein sequences for variable regions of T1AM09 and L1AM04 are GenBank: MZ600149 - MZ600152. Glycan array data were deposited in the NCBI Gene Expression Omnibus (GEO) database with accession number GSE180517.

Field-specific reporting

Please select the one below that is the best fit for your research. If you are not sure, read the appropriate sections before making your selection.

☒ Life sciences ☐ Behavioural & social sciences ☐ Ecological, evolutionary & environmental sciences

For a reference copy of the document with all sections, see nature.com/documents/nr-reporting-summary-flat.pdf

Life sciences study design

All studies must disclose on these points even when the disclosure is negative.

Sample size	We selected samples from our cohort with available PBMC and high anti-AM titers.
Data exclusions	No data were excluded from the analysis
Replication	All attempts of replication have been successful
Randomization	NA
Blinding	NA

Reporting for specific materials, systems and methods

We require information from authors about some types of materials, experimental systems and methods used in many studies. Here, indicate whether each material, system or method listed is relevant to your study. If you are not sure if a list item applies to your research, read the appropriate section before selecting a response.

Materials & experimental systems

n/a	Involved in the study	
☐	☒	Antibodies
☐	☒	Eukaryotic cell lines
☒	☐	Palaeontology and archaeology
☐	☒	Animals and other organisms
☐	☒	Human research participants
☐	☒	Clinical data
☒	☐	Dual use research of concern

Methods

n/a	Involved in the study	
☒	☐	ChIP-seq
☐	☒	Flow cytometry
☒	☐	MRI-based neuroimaging

Antibodies

Antibodies used	Goat anti-human IgG HRP 2048-05 I1911-RJ35G Southern Biotech<br>Goat anti-human IgG HRP 2048-05 B0819-Q380B Southern Biotech<br>TRITC anti-Human IgG 2040-03 K0211-MD587 Southern Biotech<br>Surelight P3 Cy5 D7-2212 P3001-09-029 Columbia Biosciences<br>Rabbit anti-human IgG (H+L), Mouse ads-UNLB 6145-01 Southern Biotech<br>Goat anti-human IgG-Biotin 209-065-098 118179 Jackson Immunoresearch<br>FITC anti-human CD3 (B209500) Biolegend<br>PE Streptavidin (B243518) Biolegend<br>PE/Cy7 anti-human CD19 (B242979) Biolegend<br>FITC anti-human CD14 (B247208) Biolegend<br>APC anti-human IgG Fc (B232398) Biolegend
Validation	All antibodies used in this study were verified for use for ELISA, Fluorescent Microscopy, or Flow Cytometry by the manufacturer. Secondary antibodies were titrated for optimal performance.

Eukaryotic cell lines

Policy information about cell lines

Cell line source(s)	293T and F cell lines were used for protein expression and obtained as a generous gift from Dr. Kartik Chandran and Dr. Jonathan Lai at Albert Einstein College of Medicine.
Authentication	The cell lines used were authenticated

Mycoplasma contamination	Cell lines were not tested for mycoplasma contaminaton
Commonly misidentified lines (See ICLAC register)	*Name any commonly misidentified cell lines used in the study and provide a rationale for their use.*

Animals and other organisms

Policy information about studies involving animals; ARRIVE guidelines recommended for reporting animal research

Laboratory animals	C57BL/6 female mice (7–8 weeks old; Charles River)
Wild animals	The study did not involve wild animals
Field-collected samples	The study did not involve field-collected samples
Ethics oversight	The animal studies were approved by the Institutional Animal Care and Use Committee at the Albert Einstein College of Medicine.

Note that full information on the approval of the study protocol must also be provided in the manuscript.

Human research participants

Policy information about studies involving human research participants

Population characteristics	Both human subjects selected for this study were healthy adults with confirmed Mtb infection/exposure
Recruitment	These subjects were recruited as part of an ongoing TB immunology study. These subjects were selected because of available PBMC and high antibody titers to our antigen of interest.
Ethics oversight	The study was approved by the institutional review board of the Albert Einstein College of Medicine. All human subjects gave written informed consent before enrollment.

Note that full information on the approval of the study protocol must also be provided in the manuscript.

Clinical data

Policy information about clinical studies

All manuscripts should comply with the ICMJE guidelines for publication of clinical research and a completed CONSORT checklist must be included with all submissions.

Clinical trial registration	NA; this is not a clinical trial
Study protocol	Einstein IRB protocol #2006-428; CSU IRB approval numbers 15-104B and 09-006B
Data collection	NA
Outcomes	NA

Flow Cytometry

Plots

Confirm that:

☒ The axis labels state the marker and fluorochrome used (e.g. CD4-FITC).

☒ The axis scales are clearly visible. Include numbers along axes only for bottom left plot of group (a 'group' is an analysis of identical markers).

☒ All plots are contour plots with outliers or pseudocolor plots.

☒ A numerical value for number of cells or percentage (with statistics) is provided.

Methodology

Sample preparation	Human PBMC isolated by Ficoll separation
Instrument	Becton Dickinson FACSAria high-speed cell sorter
Software	FlowJo was used to analyze the software
Cell population abundance	We detect about 1.5-2% antigen positive B cells and sort into individual wells,
Gating strategy	Lymphocytes were first identified (FSC/SSC), then memory B cells were further enriched based on CD20+/CD19+ and CD27+. Secondary staining using streptavidin labeled with phycoerythrin (PE) was used to detect the AM-positive population.

Fluorescence-minus-one (FMO) controls were used to set the sorting gates for double positive (AM+, IgG+) B cells. More details are provided in the methods and supplemental figures.

☒ Tick this box to confirm that a figure exemplifying the gating strategy is provided in the Supplementary Information.
